# Supplementary material for: Hyperparameter tuned deep learning-driven medical image analysis for intracranial hemorrhage detection
Source: PLoS One. 2025 Jul 28;20(7):e0326255. doi: 10.1371/journal.pone.0326255 (PMC12303355; doi:10.1371/journal.pone.0326255)
Supplement: Attachment — Submitted filename: Response to Reviewer Comments_Rev03.docx [file pone.0326255.s003.docx]

**Response to Reviewer Comments**

We would like to thank the reviewers and editors for providing an opportunity to revise the manuscript. We have considered the comments carefully and revised the manuscript. The revised text is indicated in red font color.

**Review Comments:**

1. Comments to the Author: If the authors have adequately addressed your comments raised in a previous round of review and you feel that this manuscript is now acceptable for publication, you may indicate that here to bypass the “Comments to the Author” section, enter your conflict of interest statement in the “Confidential to Editor” section, and submit your "Accept" recommendation

Reviewer #1: All comments have been addressed

Reviewer #3: (No Response)

Thanks for the comment. Since all previous concerns have been addressed, I believe the manuscript is now appropriate for publication.

2. Is the manuscript technically sound, and do the data support the conclusions?

The manuscript must describe a technically sound piece of scientific research with data that supports the conclusions. Experiments must have been conducted rigorously, with appropriate controls, replication, and sample sizes. The conclusions must be drawn appropriately based on the data presented.

Reviewer #1: Yes

Reviewer #3: Yes

Thanks for the comment. The manuscript is technically sound, and the data presented sufficiently assist the conclusions drawn.

3. Has the statistical analysis been performed appropriately and rigorously?

Reviewer #1: Yes

Reviewer #3: Yes

Thanks for the comment. The statistical analysis is performed appropriately and rigorously, as confirmed by both reviewers.

4. Have the authors made all data underlying the findings in their manuscript fully available?

The PLOS Data policy requires authors to make all data underlying the findings described in their manuscript fully available without restriction, with rare exception (please refer to the Data Availability Statement in the manuscript PDF file). The data should be provided as part of the manuscript or its supporting information, or deposited to a public repository. For example, in addition to summary statistics, the data points behind means, medians and variance measures should be available. If there are restrictions on publicly sharing data—e.g. participant privacy or use of data from a third party—those must be specified.

Reviewer #1: Yes

Reviewer #3: Yes

Thanks for the comment. We have made all underlying data fully available as required by the PLOS Data policy.

5. Is the manuscript presented in an intelligible fashion and written in standard English?

PLOS ONE does not copyedit accepted manuscripts, so the language in submitted articles must be clear, correct, and unambiguous. Any typographical or grammatical errors should be corrected at revision, so please note any specific errors here.

Reviewer #1: Yes

Reviewer #3: Yes

Thanks for the comment. The manuscript is presented clearly and written in standard English, with no significant errors identified.

6. Review Comments to the Author: - Please use the space provided to explain your answers to the questions above. You may also include additional comments for the author, including concerns about dual publication, research ethics, or publication ethics. (Please upload your review as an attachment if it exceeds 20,000 characters)

Reviewer #1: The authors have made all the changes required and the manuscript can be published in the present form

Reviewer #3: The issues are listed in the following: - The professional English editing is recommended. The authors should get editing help from someone with full professional proficiency in English.

As per the reviewer’s comment, we have thoroughly investigated the paper content and removed the grammatical and typographical mistakes and removed inappropriate and redundant sentences in the revised manuscript.

7. The introduction explains the severity of intracranial hemorrhage and the importance of computed tomography (CT) in diagnosis, but it is recommended to supplement specific clinical data or cases. The introduction of some important methods would introduce high quality documents, such as CNN "DOI10.1109 / TFUZZ. 2024.3369944", "DOI10.3389 / fnagi. 2022.908143; "DOI10.1016/j.com com. 2019.11.015" bayesian method.

Based on the reviewer’s comment, we have improved the introduction section by utilizing the given citations and also made sure that the section provided sufficient discussion on the study background, motivation, study contribution, and novelty in the revised manuscript. Kindly refer to Page 2, Section 1, Paragraph 1-4.

8. It is suggested to supplement a comparative analysis to clarify the unique advantages and innovative aspects of using deep learning methods in intracranial hemorrhage detection in this research, and to explain the differences from previous medical imaging analysis tasks.

As per the reviewer’s comment, we have clearly highlighted the superiority of the proposed model over existing techniques by emphasizing the comparison study; and also made appropriate discussion on each model under the performance validation section in the revised manuscript. Please refer to Page 23, Paragraph 2; Page 24, Table 4, Fig. 10.

9. It is suggested to conduct a more detailed analysis and comparison of the core innovations, applicable scenarios, and performance of each method.

Based on the reviewer’s comment, we have added recent and sufficient citations for the comparison study under the experimental validation section; and also provided needful discussion on the comparison under the section in the revised manuscript. Kindly refer to Page 23, Paragraph 2; Page 24, Table 4, Fig. 10.

[1] Ragab, M., Salama, R., Alotaibi, F.S., Abdushkour, H.A. and Alzahrani, I.R., 2023. Political Optimizer with Deep Learning Based Diagnosis for Intracranial Hemorrhage Detection. *IEEE Access*.
[2] Nawabi, J., Schulze-Weddige, S., Baumgärtner, G.L., Orth, T., Orco, A.D., Morotti, A., Mazzacane, F., Kniep, H., Hanning, U., Scheel, M. and Fiehler, J., 2025. End-to-End Machine Learning based Discrimination of Neoplastic and Non-neoplastic Intracerebral Hemorrhage on Computed Tomography. *Informatics in Medicine Unlocked*, p.101633.

10. When introducing related work, the differences and advantages of this study compared to existing work should be more clearly pointed out.

As per the reviewer’s comment, we have thoroughly discussed the merits, demerits, and research gap of the existing studies under the literature survey section in the revised manuscript. Please refer to Page 6, Paragraph 2.

11. It is suggested to supplement relevant experiments or analyses to explain the specific application scenarios of median filtering in this study and optimization strategies.

Based on the reviewer’s comment, we have improved the introduction section by clearly stating the problem statement, motivation, study contribution, and novelty; also, added the given citations under the related works section, and made sure that the section provided needful discussion on each and every citation in the revised manuscript. Kindly refer to Page 2, Section 1, Paragraph 1-4; Page 4, Section 2, Paragraph 1-3.

12. The basic structure and improvement methods of EfficientNet are introduced in detail, but there is insufficient information on specific parameter settings and optimizer selection in the model training process.

Thanks for the comment. To optimize the GECM-EfficientNet model, specific parameter settings and optimizer choices play a significant role in training efficiency. The model employs the Adam optimizer with a learning rate of 0.001, chosen for its capability to adapt the learning rate during training and handle sparse gradients. The batch size is set to 32, and early stopping is applied to prevent overfitting, with a patience of 10 epochs. The learning rate of the model is additionally fine-tuned utilizing a learning rate scheduler that mitigates the rate after each epoch based on performance. Furthermore, weight decay is applied to regularize the model and prevent overfitting. The training process integrates transfer learning by initializing the EfficientNetB0 model with pre-trained weights from ImageNet, followed by fine-tuning on the target waste image dataset for improved feature extraction.

13. In the analysis of experimental results, it is necessary to more detailedly explain the basis for selecting evaluation metrics.

Thanks for the comment. The proposed HPDL-MIAIHD approach employed metrices namely $Accu_{y}$, $Prec_{n}$, $Reca_{l}$, $F_{score}$, and MCC for the analysis process. The metrices are chosen for their overall analysis capabilities in computing the HPDL-MIAIHD approach. $Accu_{y}$ provides a comprehensive measure of correct predictions, while $prec_{n}$ and $reca_{l}$ concentrate on reducing false positives and detecting all relevant positives, subsequently. The $F_{score}$ balances $prec_{n}$ and $reca_{l}$, giving an integrated metric of their performance. Furthermore, MCC computes the quality of binary classifications, considering true and false positives and negatives, making these metrics substantial for robustly analyzing the HPDL-MIAIHD technique across varied performance factors and ensuring reliable model evaluation.

14. The conclusion summarizes the accuracy of the HPDL-MIAIHD method, but the analysis of the challenges encountered in the research process, the methods for solving problems, and the limitations of the method are not in-depth enough.

As per the reviewer’s comment, we have employed a distinct sub-section for discussing the limitations and future works of the proposed model under the conclusion section in the revised manuscript. Please refer to Page 27, Section 5.1, Paragraph 1-2.

15. It is suggested to combine the limitations of the current research and clinical needs to propose more targeted and feasible research directions.

Based on the reviewer’s comment, we have thoroughly discussed the limitations and future works of the proposed model under a separate section in the revised manuscript. Kindly refer to Page 27, Section 5.1, Paragraph 1-2.
