# Supplementary material for: Hyperparameter tuned deep learning-driven medical image analysis for intracranial hemorrhage detection
Source: PLoS One. 2025 Jul 28;20(7):e0326255. doi: 10.1371/journal.pone.0326255 (PMC12303355; doi:10.1371/journal.pone.0326255)
Supplement: Attachment — Submitted filename: Response to Reviewer Comments.docx [file pone.0326255.s002.docx]

**Response to Reviewer Comments**

We would like to thank the reviewers and editors for providing an opportunity to revise the manuscript. We have considered the comments carefully and revised the manuscript. The revised text is indicated in red font color.

**Review Comments: 1**

1. We've checked your submission and before we can proceed, we need you to address the following issues:

1. Are these third party data (i.e., data not owned or collected by the author(s))?

2. If these are indeed third party data, please explain how others can access these datasets and confirm that others would be able to access these data in the same manner as the authors. Please also confirm that the authors did not have any special access privileges that others would not have.

3. If these are not third party data but there are ethical or legal restrictions on sharing a de-identified data set, please explain them in detail (e.g., data contain potentially identifying or sensitive information) and who has imposed them (e.g., a governmental body, an ethics committee, etc.). Please also provide contact information for a data access committee, ethics committee, or other institutional body to which data requests may be sent.

4. If these are not third party data and there are no restrictions, please upload the minimal anonymized dataset necessary to replicate your study findings to a stable, public repository and provide us with the relevant URLs, DOIs, or accession numbers. For a list of recommended repositories, please see https://journals.plos.org/plosone/s/recommended-repositories. You also have the option of uploading the data as Supporting Information files, but we would recommend depositing data directly to a data repository if possible.

Thanks for the comment. The dataset implemented in this study is not collected by the authors but is publicly available, as cited in [34] (https://physionet.org/content/ct-ich/1.3.1/). The dataset comprises of 82 CT scans of patients with traumatic brain injury (TBI), with regions of intracranial hemorrhage (ICH) and fractures annotated by radiologists. It encompasses scans from 75 subjects in NIfTI format. This dataset is publicly accessible, and no special access privileges were provided to the authors. The dataset was collected with ethical approval from the research and ethics board in the Iraqi Ministry of Health, Babil Office (approval #1369). Please refer to Page 15, Section 4, Paragraph 1; Page 16, Table 1.
